# News

**DOI:** 10.1155/S1463924601000165

**Published:** 2001


Journal of Automated Methods & Management in Chemistry, Vol. 23, No. 4 (July–August 2001) pp. 133–134

### News

### MultiDose® automates stability study

Zymark Corporation announced that Metrics, Inc. recently purchased several Zymark MultiDose® Automated Dissolution Workstations to increase efficiency in analysing a large number of dissolution samples.

Metrics, located in Greenville, NC, USA, is a contract pharmaceutical development and analytical laboratory. MultiDose provides automated dissolution results for USP Apparatus I and II. The MultiDose also does USP Apparatus II runs with Capsule Sinkers. Each sinker weighs down a capsule, properly simulating stomach digestion and providing more accurate dissolution testing results.

When entering a large Phase III Stability Study that involved massive amounts of tablet dissolution testing, Metrics found that other automated dissolution testing products only automated one or two of the tasks being performed and instead MultiDose would completely automate dissolution testing, including the filling and cleaning of baths. Eight batches can be set to run overnight.

The MultiDose family of automated dissolution workstations provides a compliant way of achieving high levels of dissolution throughput. These systems require a minimum of operator set-up time and are designed to become productive immediately.

*For additional information contact: Tom Maltais, Zymark Corporation, Hopkinton, MA, USA. Tel: + 1 508 497 6541; e-mail: Tom.Maltais@Zymark.com*

### Announcements

### Agilent Technologies releases GC-ICP-MS Interface technology brief

Agilent Technologies Europe, announced the release of the technology brief 'Agilent GC-ICP-MS Interface' (publication no. 5988-3071EN). Agilent Technologies has combined its expertise in gas chromatography and inductively coupled plasma mass spectroscopy (ICP-MS) to introduce the first fully integrated solution for the coupling of the two techniques. The separation capabilities of gas chromatography and the high sensitivity and selectivity of the Agilent 7500 Series ICP-MS provide analysts with the capability to separate and quantitate ultratrace levels of organometallic compounds.

The measurement capability of currently available GC detectors such as FPD, FID and ECD are good, but the need for determining organometallic compounds at increasingly lower concentrations has fuelled the investigation of alternative detection systems. This measurement challenge led to the development of the Agilent GC-ICP-MS Interface. ICP-MS is being used more frequently in combination with a front-end separation technique such as GC, as a specific and highly selective detector for a variety of separation applications.

As expected with a fully integrated system, the interface between the GC and the ICP-MS is seamless and allows for the simultaneous separation and measurement of multiple organometallic compounds in a single analytical run.

*For more information contact: Agilent Technologies Europe, PO Box 18324, London EC2M 5XA, UK*

### New research shows how to predict consequences of chemical spills

The Health and Safety Executive (HSE) has published a research report that describes a new theoretical model that can predict the consequences of chemical spills. The report finds that existing models cannot satisfactorily describe the outcome of reactive chemicals on contact with water. The behaviour of such substances after spillage is complex, and the effects on people and the environment are highly variable.

A spreading liquid chemical pool will react with water on the ground and sometimes with moisture in the atmosphere. The reaction will generate heat, raising the liquid temperature and increasing the amount of vapour.

The new model 'REACTPOOL' describes the pool behaviour of water reactive chemicals in a realistic way. It was developed from previous work on accidental releases of water reactive sulphur trioxide and oleum. The results of applying the model indicate that pool behaviour is principally affected by the way the chemical reacts with water, the amount of water available, surface roughness and wind speed.

